# Preventive Effect of Anji White Tea Flavonoids on Alcohol-Induced Gastric Injury through Their Antioxidant Effects in Kunming Mice

**DOI:** 10.3390/biom9040137

**Published:** 2019-04-04

**Authors:** Bihui Liu, Xingxing Feng, Jing Zhang, Yang Wei, Xin Zhao

**Affiliations:** 1 Chongqing Collaborative Innovation Center for Functional Food, Chongqing University of Education, Chongqing 400067, China; liubh@foods.ac.cn (B.L.); grace951006@163.com (Y.W.); 2 Chongqing Engineering Research Center of Functional Food, Chongqing University of Education, Chongqing 400067, China; 3 Chongqing Engineering Laboratory for Research and Development of Functional Food, Chongqing University of Education, Chongqing 400067, China; 4 College of Biological and Chemical Engineering, Chongqing University of Education, Chongqing 400067, China; fengxx@foods.ac.cn; 5 Environment and Quality Inspection College, Chongqing Chemical Industry Vocational College, Chongqing 401228, China; zhangjing@foods.ac.cn

**Keywords:** Anji white tea, flavonoid, alcoholic gastric injury, messenger RNA expression, oxidation

## Abstract

Anji white tea (*Camellia sinensis*) is a traditional Chinese tea beverage, which is classified as green tea and contains an abundant amount of flavonoids. In this study, the preventive effect of Anji white tea flavonoids (AJWTFs) on ethanol/hydrochloric acid-induced gastric injury in mice was evaluated. The serum and gastric tissues of mice were analyzed using a biochemical kit and by quantitative polymerase chain reaction (qPCR). Observation of the appearance of the stomach indicated that AJWTFs could effectively reduce the area of gastric injury caused by ethanol/hydrochloric acid, and the inhibition rate of AJWTF on gastric injury increased with an increase in AJWTF concentration. The Anji white tea flavonoids could also reduce the volume and pH of gastric juice in mice with gastric injury. Biochemical results showed that AJWTFs could increase the superoxide dismutase (SOD) and glutathione (GSH) activities, as well as decrease the malondialdehyde (MDA) level, in the serum and liver of mice with gastric injury. Pathological observation confirmed that AJWTFs could inhibit the tissue damage caused by ethanol/hydrochloric acid in the stomach of mice. Further qPCR experiments also showed that AJWTFs could inhibit the decreases in neuronal nitric oxide synthase (nNOS), endothelial nitric oxide synthase (eNOS), copper/zinc superoxide dismutase (Cu/Zn–SOD), manganese superoxide dismutase (Mn–SOD), catalase (CAT), and the increase in inducible nitric oxide synthase (iNOS) expression in the gastric tissue of mice caused by gastric injury. As observed, AJWTFs exerted a good preventive effect on alcohol-induced gastric injury in mice induced by ethanol/hydrochloric acid, and the effect is close to that of ranitidine. Anji white tea flavonoids present good antioxidant effect, which allows them to effectively prevent alcoholic gastric injury and be used as biologically active substances with a broad range of applications.

## 1. Introduction

Alcoholic gastric injury is a gastric mucosal injury caused by excessive drinking, which often clinically manifests as gastritis. Ethanol can directly damage gastric epithelial cells and submucosal vessels [1], causing direct injury to the epithelium of the gastric mucosa. Ethanol directly damages the epithelial cells in the gastric mucosa, destroying the barrier of the gastric mucosa. Hydrophobic alkyl groups and hydrophilic hydroxyl groups of ethanol molecules can directly destroy the defense system of the gastric mucosa, rendering the gastric mucosa vulnerable to various digestive enzymes, bile, and gastric acid. This susceptibility leads to H^+^ antidiffusion, thereby aggravating the damage to the gastric mucosa. Ethanol can cause injuries to the submucosal vascular endothelium, hemangiectasia, small blood vessel bursts, submucosal hemorrhage, and other changes, further destroying the mucosal barrier. Alcohol can also cause neutrophils to infiltrate and further aggravate mucosal damage as a result of numerous inflammatory mediators produced by mucosal epithelial and vascular endothelial damage [2]. In addition, increased ethanol concentration exerts a strong stimulating effect on the gastric mucosa and can cause necrosis of mucosal epithelial cells [3]. The frequent occurrence of gastritis, gastric ulcer, gastric perforation, gastric bleeding, and other gastric diseases recently directed the attention toward alcoholic gastric injury. In addition to drug treatment, the preventive effect and application of bioactive substances on alcoholic gastric injury also has research interest.

White tea is a kind of micro-fermented tea, which is one of the six major tea categories in China. Most white tea is unique, mainly because the production process is very simple, mainly including four processes of picking, spreading, withering, and drying. Anji white tea (AJWT) is mainly produced in Anji County, Zhejiang Province, China. It is called white tea because its raw tea leaves are white as a result of chlorophyll deficiency, which is attributed to variation. However, AJWT varies from other white tea in its processing technology. It is non-fermented tea and is classified as green tea; it is made through six processes (picking, spreading, killing, sorting, drying, and preservation), unlike other white tea [4]. Many bioactive compounds are found in flavonoids, which can prevent and treat cardiovascular and cerebrovascular diseases by reducing the fragility of blood vessels, improving the permeability of blood vessels, reducing blood lipids and cholesterol, preventing and treating hypertension, cerebral hemorrhage, coronary heart disease, and angina pectoris, expanding coronary vessels, and increasing coronary flow [5,6]. Simultaneously, flavonoids protect and detoxify the liver and act as an antifungal [7]. Most physiological activities of plant flavonoids are based on their antioxidant effects [8]. In vitro studies showed that Anji white tea flavonoids (AJWTFs) exert scavenging effects on superoxide anion radicals (O_2_^•−^), hydroxyl radicals (^•^OH), 2,2-diphenyl-1-picrylhydrazyl (DPPH), and hydrogen peroxide [9]. Therefore, AJWTFs are also substances with antioxidant activity, which may benefit and protect the body. Several studies also indicated that flavonoids from natural plants exert a certain inhibitory effect on gastric injury [10,11,12]. Flavonoids in natural plants can inhibit gastric injury through their antioxidant effects [11,12]. There are few studies on AJWTFs, whereby only in vitro studies showed that AJWTFs have the ability to scavenge superoxide anion free radicals, hydroxyl free radicals, hydrogen peroxide, and DPPH free radicals [13]. Accordingly, the protective effect of AJWTFs on the stomach needs to be investigated.

Few studies on AJWTFs were conducted, and the understanding and application of active substances in this natural beverage are inadequate. In this study, a mouse model of alcoholic gastric injury was established to observe the preventive effect of AJWTFs on alcoholic gastric injury in animals; in addition, the mechanism of action of AJWTFs on oxidative stress induced by gastric injury in mice was observed. Anji white tea is currently only used as a common beverage. Serum and tissues in mice were observed by biochemical detection and based on experiments in molecular biology. In accordance with the results of this study, ideas are proposed for the further application of AJWT, particularly the mechanism of the AJWTF extract, to accumulate theoretical evidence of the application of AJWTFs as functional foods with biological activity. This study only focused on experimental animals; regardless, it lays the foundation for further clinical research on AJWTF.

## 2. Materials and Methods

### 2.1. Extraction of Anji White Tea Flavonoids

Anji white tea leaves was picked in Anji County, Zhejiang Province, China in mid-March 2018. Tea canopy surface reached 10–15 standard buds per square meter for picking. Tea trees were picked one leaf per bud according to the requirements. Up to 1 kg of freeze-dried AJWT leaf samples was weighed and powdered. Anji white tea leaves powders were divided into 10 parts on average. One liter of ethanol solution with a concentration of 70% (v/v) was added to each part. After extraction for 4 h at 70 °C in a water bath, 10 filtrates were collected and filtered by diatomite to remove lipid-soluble impurities. All extracts were collected again and then added to the column containing ADS-17 resin. AJWTF were adsorbed by the resin. Subsequently, 90% (v/v) ethanol was used to elute the resin and dissolve the adsorbed flavanone in ethanol. The AJWTF extract was ultimately obtained by steam-drying ethanol [14].

### 2.2. Determination of Anji White Tea Flavonoid Content

A specific amount (10 mg) of rutin standard (Hefei Bomei Biotechnology Co., Ltd., Hefei, China) was added into a 25-mL volumetric flask; 10 mL of (*v*/*v*) 90% ethanol was added, followed by 0.75 mL of 5% NaNO_2_ solution, 0.75 mL of 10% Al(NO_3_)_3_ solution, and 10 mL of 4% NaOH solution. A rutin standard solution in 90% ethanol was ultimately prepared, and 10, 20, 30, 40, and 50 μg/mL rutin concentrations were determined at 500 nm (Evolution 300 ultraviolet spectrophotometer, Thermo Fisher Scientific, Inc., Waltham, MA, USA). The absorbance of the standard solution and the rutin standard curve were calculated. Subsequently, 0.01 g of AJWTF extract was dissolved in 5 mL of 90% ethanol, and 2 mL of AJWTF extract solution was poured into a 25-mL volumetric flask. The flavonoid content in the AJWTF extract was determined using the repeated rutin standard curve method (rutin meter) [14].

### 2.3. Establishment of Alcohol-Induced Gastric Injury Model in Mice

Specific pathogen-free (SPF) Kunming mice aged 6 weeks (male, body weight 20 ± 2 g) were purchased from Chongqing Medical University and fed with mouse basic feed and drinking water; the bedding material was changed once every two days for one week. The mice were divided into five groups with 10 mice each: the normal group, model group, low concentration AJWTF group (AJWTF-L group), high concentration AJWTF group (AJWTF-H group), and ranitidine group. The normal and model groups were given normal saline by gastric perfusion; the AJWTF-L and AJWTF-H mice were given 100 and 200 mg/kg AJWTF via gastric perfusion, respectively; the ranitidine group was given 50 mg/kg ranitidine for 14 days via gastric perfusion, and all experimental mice were fasted for 24 h on day 14. On day 15, all mice, except for those in the normal group, received an ethanol mixture (60% ethanol, 40% 150 mmol/L hydrochloric acid) 1 h after gastric administration, with 0.1 mL of ethanol mixture per 10 g of mouse body weight [15]. After intragastric administration for 30 min, blood from the eyeball was collected, and gastric tissue was dissected for use. The volume and pH of the gastric juice were measured (pH meter, PHS-25, Shanghai INESA Scientific Instrument Co., Ltd., Shanghai, China), while the degree of gastric mucosal injury was assessed intuitively. The inhibitory rate of gastric injury (%) = (1 − area of gastric injury/area of gastric tissue) × 100% was calculated using the inhibitory rate of the gastric injury after pictures were taken. The protocol for these experiments was approved by the Animal Ethics Committee of Chongqing Collaborative Innovation Center for Functional Food (201802003B) on February 22, 2018.

### 2.4. Determination of Superoxide Dismutase and Glutathione Activities and Malondialdehyde Level in Serum

Blood was kept at room temperature for 1 h; then, the blood was centrifuged at 4000 rpm for 10 min, the upper limit of the serum was measured, and the superoxide dismutase (SOD), glutathione (GSH), and malondialdehyde (MDA) levels in the mouse serum were determined in accordance with the instructions provided in the superoxide dismutase (SOD) assay kit (WST-1 method), glutathione (GSH) assay kit (spectrophotometric method) and malondialdehyde (MDA) assay kit (TBA method) (Nanjing Jiancheng Bioengineering Institute, Nanjing, Jiangsu, China) [16].

### 2.5. Determination of Superoxide Dismutase and Glutathione Activities and Malondialdehyde Level in Gastric Tissue

Mouse liver was used to prepare 10% homogenate, which was then centrifuged at 4000 rpm for 10 min. The supernatant was taken out, and the SOD, GSH, and MDA levels in gastric tissues were determined in accordance with the instructions accompanying the kits (Nanjing Jiancheng Bioengineering Institute, Nanjing, Jiangsu, China) [16].

### 2.6. Pathological Observation of Gastric Tissue

About 0.5 cm^2^ mouse gastric tissue was immobilized in 10% formalin solution for 48 h. The gastric tissue was dehydrated, transparent, waxed, embedded, and sectioned. Hematoxylin and eosin staining was performed to observe the morphological changes under an optical microscope.

### 2.7. Quantitative Polymerase Chain Reaction Assay

Gastric tissues of mice were crushed, and total RNA was extracted from the gastric tissues with the use of RNAzol. The concentration of the total RNA was then diluted to 1 μg/μL. The diluted total RNA solution of 5 μL was then taken out, and the reverse transcription kit was used to obtain the template of the DNA. Then, 2 μL of DNA template was mixed with 10 μL of SYBR Green PCR Master Mix and primers (1 μL of forward and 1 μL of reverse), and then reacted at 95 °C for 60 s. Forty cycles were performed at 95 °C for 15 s, 55 °C for 30 s, and 72 °C for 35 s. Gene expression (Table 1) was then detected at 95 °C for 30 s and 55 °C for 35 s. Glyceraldehyde-3-phosphate dehydrogenase (GAPDH) was used as the internal reference, and the relative expression of the gene was calculated using the 2^−ΔΔ^^Ct^ method [17].

### 2.8. Statistical Analysis

Three parallel experiments were conducted on the serum and tissue indexes of each mouse, and the average values were obtained. The data were analyzed using the SAS 9.1 statistical software (SAS Institute, Inc., Cary, NC, USA) [13]. One-way analysis of variance (ANOVA) was used to analyze whether significant differences existed among the groups at the level of *p* < 0.05.

## 3. Results

### 3.1. Content of Anji White Tea Flavonoid Extracts

The regression equation of the standard curve of the rutin standard solution was *Y* = 842.34*X* + 0.1509, where *Y* is the concentration of the rutin standard solution, and *X* is the absorbance value. According to the standard curve calculation, the purity of flavonoids in AJWTF extracts reached 68.3% (rutin calculation), which proved that flavonoids were the main components in the follow-up animal experiments.

### 3.2. Volume and pH of Gastric Juice in Mice

Table 2 shows that the model group exhibited the largest gastric juice volume and the lowest pH, whereas the normal group exhibited the lowest volume and the highest pH. Both the AJWTF and ranitidine decreased the volume and increased the pH of gastric juice in mice with gastric injury, relative to those in the model group. The effect of AJWTF-H was similar to that that of ranitidine; that is, AJWTF-H and ranitidine could significantly (*p* < 0.05) reduce gastric juice volume and increase gastric juice pH in mice with gastric injury compared with AJWTF-L. Anji white tea flavonoids could effectively reduce the increase in volume and decrease in pH of gastric juice as a result of gastric injury.

### 3.3. Gastric Damage Area and Inhibitory Rate of Anji White Tea Flavonoids in Mice

**Figure 1:**
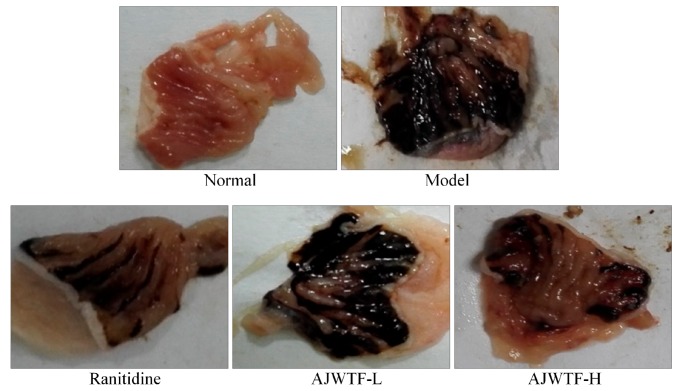
Stomach specimens of mice from each group. Ranitidine: Ranitidine at 50 mg/kg body weight (b.w.) by gavage; AJWTF-L: Anji white tea flavonoids at 100 mg/kg b.w. by gavage; AJWTF-H: Anji white tea flavonoids at 200 mg/kg b.w. by gavage.

Figure 1 and Table 3 show that ethanol causes a large area of gastric mucosal damage. Anji white tea flavonoids and ranitidine could significantly reduce the area of gastric mucosal damage (*p* < 0.05). The effect of AJWTF-H was stronger than that of AJWTF-L but slightly weaker than that of ranitidine. Owing to the AJWTF and ranitidine, the inhibition rate of AJWTF-H on gastric mucosal injury was also significantly higher than that of AJWTF-L (*p* < 0.05) and slightly lower than that of ranitidine. Anji white tea flavonoids could effectively reduce the area of alcohol-induced gastric mucosal damage and the degree of gastric injury.

### 3.4. Pathological Observation of Gastric Tissue in Mice

**Figure 2:**
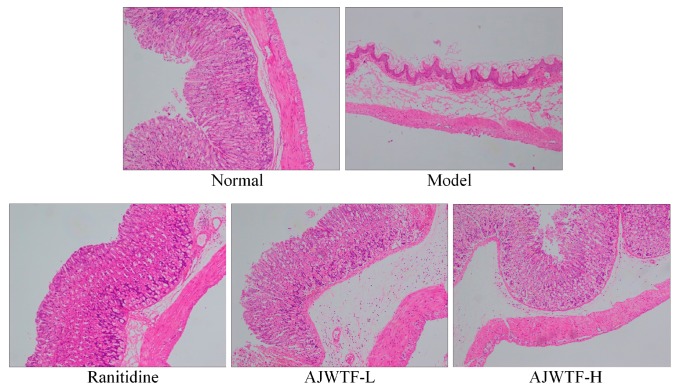
Hematoxylin and eosin-stained stomach sections of mice from each group (100×). Ranitidine: Ranitidine at 50 mg/kg body weight (b.w.) by gavage; AJWTF-L: Anji white tea flavonoids at 100 mg/kg b.w. by gavage; AJWTF-H: Anji white tea flavonoids at 200 mg/kg b.w. by gavage.

As shown in Figure 2, the structure of gastric tissue in normal mice was intact, the cells were tightly arranged and orderly, the cells were of the same size, and the surface epithelium was intact and did not fall off. In the model group, the gastric tissue structure was incomplete, the number of cells decreased markedly, and the arrangement of cells was completely disrupted. The upper epidermis was exfoliated, and severe hemorrhage between the cells and tissues was observed. In the AJWTF-L group, the gastric cells were inconsistent in size and loosely arranged; part of the cell was damaged, and those with hyperemia and hemorrhage were exfoliated to a certain extent. Compared with the AJWTF-L mice, the AJWTF-H mice showed a more normal cell arrangement and tightness, more complete upper epidermis, and less congestion and hemorrhage. In the ranitidine group, the cells in gastric tissue were tightly arranged, and only individual cells appeared scattered; mucosal exfoliation was not obvious, and the cells and tissues were only slightly congested. The results indicated that AJWTF could protect gastric tissues and prevent ethanol-induced damage; in addition, a large dose was better than a small dose.

### 3.5. Superoxide Dismutase and Glutathione Activities and Malondialdehyde Level in Mouse Serum and Gastric Tissues 

Table 4 and Table 5 show that the model group exhibited the lowest SOD and GSH activities and the highest MDA level in gastric tissues. The gastric tissues of mice in the normal group showed the opposite trend. The SOD and GSH activities in gastric tissues were the highest, whereas the MDA levels were the lowest. Anji white tea flavonoids could significantly increase the SOD and GSH activities in the gastric tissue of mice with gastric injury (*p* < 0.05) and decrease the MDA level (*p* < 0.05). The higher the AJWTF concentration was, the more obvious the effect and the closer it was to that of ranitidine.

### 3.6. Expression of nNOS, eNOS, and iNOS Messenger RNA in Gastric Tissue of Mice

**Figure 3:**
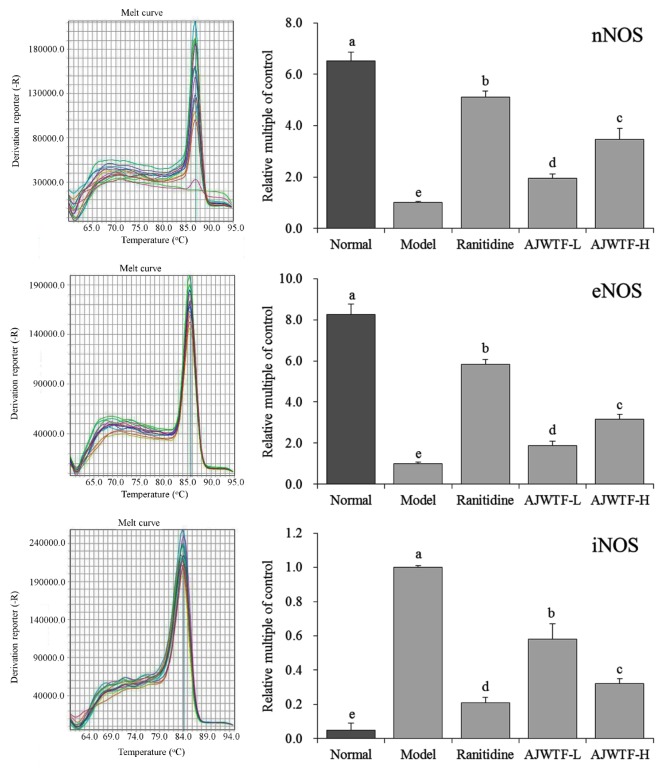
Messenger RNA expression of neuronal nitric oxide synthase (nNOS), endothelial nitric oxide synthase (eNOS), and inducible nitric oxide synthase (iNOS) in the stomach tissues of mice from each group. Values presented are the means ± standard deviation. ^a–^^c^ Mean values with different letters in the same bars indicate significant difference (*p* < 0.05) according to Duncan’s multiple-range test. Ranitidine: Ranitidine at 50 mg/kg body weight (b.w.) by gavage; AJWTF-L: Anji white tea flavonoids at 100 mg/kg b.w. by gavage; AJWTF-H: Anji white tea flavonoids at 200 mg/kg b.w. by gavage.

Figure 3 shows that the expression levels of neuronal nitric oxide synthase (nNOS), endothelial nitric oxide synthase (eNOS), and inducible nitric oxide synthase (iNOS) were lowest in the model group. After AJWTF treatment, the expression levels of nNOS and eNOS in gastric tissues of mice with gastric injury increased significantly (*p* < 0.05), whereas that of iNOS decreased significantly (*p* < 0.05). The effect of AJWTF-H was stronger than that of AJWTF-L and similar to that of ranitidine.

### 3.7. Expression of Cu/Zn–Superoxide Dismutase, Mn–Superoxide Dismutase, and Catalase Messenger RNA in Gastric Tissues of Mice

**Figure 4:**
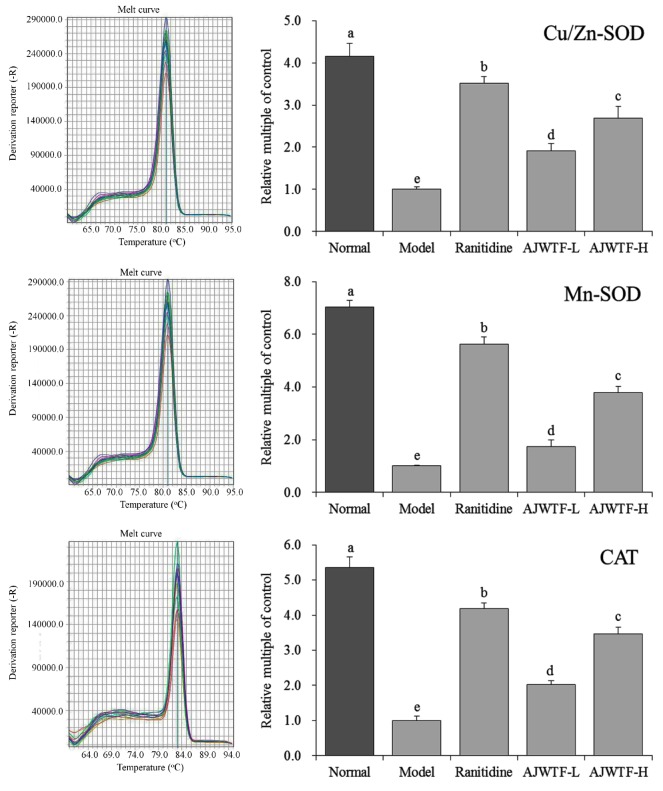
Messenger RNA expression of copper/zinc superoxide dismutase (Cu/Zn–SOD), manganese SOD (Mn–SOD), and catalase (CAT) in the stomach tissues of mice from each group. Values presented are the means ± standard deviation. ^a–^^c^ Mean values with different letters in the same bars indicate significant difference (*p* < 0.05) according to Duncan’s multiple-range test. Ranitidine: Ranitidine at 50 mg/kg body weight (b.w.) by gavage; AJWTF-L: Anji white tea flavonoids at 100 mg/kg b.w. by gavage; and AJWTF-H: Anji white tea flavonoids at 200 mg/kg b.w. by gavage.

Figure 4 shows that the highest expression of Cu/Zn–SOD, Mn–SOD, and catalase (CAT) was observed in the liver tissues of mice in the normal group. The expression of Cu/Zn–SOD, Mn–SOD, and CAT in gastric tissues was significantly reduced by ethanol-induced gastric injury (*p* < 0.05). Both AJWTF and ranitidine could significantly inhibit the expression of Cu/Zn–SOD, Mn–SOD, and CAT in ethanol-induced gastric tissue injury (*p* < 0.05). With increasing concentration, AJWTF increased the expression of C/Zn–SOD, Mn–SOD, and CAT in gastric tissues. The higher the expression of Cu/Zn–SOD, Mn–SOD, and CAT was, the closer the effect exerted by AJWTF-H was to that of ranitidine.

## 4. Discussion

The alcohol-induced gastric injury model is used to test human gastric acid secretion and simulate alcohol-induced acute digestive injury. Modeling of the damage helps assess whether potential bioactive substances exert protective and antioxidant effects on gastric tissues [18]. Ethanol/hydrochloric acid solution can be used to observe the ulceration of gastric mucosa in mice after gastric administration. The area of ulceration can be used to directly determine the degree of gastric injury by using related indicators [19]. 

Unlike infectious diseases, gastric injury is multifactorial and develops through different contacts. Different risk factors and unhealthy lifestyle are identified as the main causes. Ethanol is regarded as an important factor inducing human gastric injury mainly because the gastric mucosa is easy to penetrate and destroy, exposing it to gastric acid and pepsinase [20]. Under the combined action of gastric acid and pepsinase, severe vasoconstriction can occur within a short time, accompanied by rapid dilation of small arteries. These reactions of blood vessels promote vascular injury and ultimately lead to the formation of gastric injury [21]. In the current study, AJWTF could effectively reduce the area of ethanol/hydrochloric acid-induced gastric injury and protect the gastric mucosa. The inhibition rate of gastric injury in the AJWTF group was similar to that in the normal group and the ranitidine group, indicating that AJWTF prevented the gastric injury to a certain extent. 

Normally, gastric juice does not affect the gastric mucosa. After gastric mucosal injury, the stomach is stimulated and secretes a large amount of gastric acid, thus increasing the volume of gastric juice in the stomach and decreasing the pH of gastric juice. These effects aggravate the damage to the gastric mucosa, exacerbating the gastric injury, which then leads to a vicious circle [22]. In the current study, AJWTF could effectively decrease the volume of gastric juice and increase the pH of gastric juice in mice with gastric injury, thus protecting the gastric tissues and alleviating alcoholic gastric injury. 

Meanwhile, nNOS, eNOS, and iNOS represent the neuronal type, endothelial type, and inducible type of NOS, respectively. Nitric oxide synthase is the rate-limiting enzyme of NO synthesis and widely exists in the normal tissues of humans and animals [23]. Under normal physiological conditions, the mechanism of NO production, release, diffusion, and inactivation is precisely regulated in the nervous system, which is mainly achieved by regulating the activation and deactivation of nNOS [24]. Neuronal NOS not only plays an important role in the nervous system but is also distributed in gastric mucosal epithelial cells. Neuronal NOS regulates blood flow and muscle contraction by regulating NO to control the degree of gastric injury [25]. The decrease in nNOS expression can aggravate gastric tissue damage [26]. The expression and activity of eNOS are relatively stable. Nitric oxide derived from eNOS mainly participates in promoting epithelial repair, regulating gastric mucosal blood flow and adaptive cell protection, inhibiting gastric acid secretion, enhancing mucus barrier function, and promoting vascular regeneration [27]. Simultaneously, eNOS can inhibit the oxidative damage of blood vessels caused by oxidative stress, as well as relax and protect blood vessels [28]. Once iNOS is activated, enzyme activity continues for an extended time and produces NO in large quantities. Low NO concentration can effectively resist gene mutation and activate the defense mechanism of the body, whereas high NO concentrations can lead to loss of control of gene mutation, thus stimulating gene mutation and aggravating tissue damage [29]. Anji white tea flavonoids can promote nNOS and eNOS expression in the gastric tissues of mice with gastric injury, as well as reduce the expression of iNOS to inhibit inflammatory response and protect gastric mucosa, thus inhibiting gastric injury. Oxidative stress is the main characteristic of gastric mucosal injury. Damage to gastric mucosal cells is aggravated by the decrease in antioxidants, such as SOD, CAT, and GSH, as well as the increase in free radicals after ethanol treatment [30]. As a response to free-radical accumulation, SOD and other cell antioxidant enzymes are considered the first line of defense against oxidative damage [31]. In addition, CAT and GSH exert similar effects, which can eventually clear free radicals in the body, maintain the balance between oxidation and antioxidation, and protect the body from free radical damage; thus, the content of antioxidant enzymes can directly reflect the number of free radicals in the body [32,33]. Cu/Zn–SOD is mainly found in the cytoplasm, whereas Mn–SOD is typically present in mitochondria. Cu/Zn–SOD and Mn–SOD sensitivity to different factors varies [34]. After ethanol-induced gastric mucosal injury, the SOD, CAT, and GSH activities in the body decrease significantly [17,35]. The reduced activity of antioxidant enzymes may trigger chain lipid peroxidation, resulting in the decreased fluidity and enhanced permeability of biofilms [36]. Malondialdehyde is produced by lipid decomposition of peroxide; thus, the MDA level is often used as a marker of lipid peroxidation and reacts as free radicals are produced [37]. In the present study, the SOD and GSH activities in the serum and gastric tissues of the AJWTF-treated mice with gastric damage were significantly higher than those of the model group. In addition, the MDA content was significantly lower in the AJWTF group than in the model group. Further experiments confirmed that AJWTF could upregulate the expression of Cu/Zn–SOD, Mn–SOD, and CAT in the gastric tissues of mice with gastric injury. The results indicated that AJWTF could reduce the free radical damage caused by ethanol and lipid peroxidation protecting the balance of antioxidant in vivo to inhibit alcohol-induced gastric injury. Rutin has a strong antioxidant effect, which can inhibit the damage and aggravation of gastric mucosa caused by oxidative stress [38]. At the same time, rutin can inhibit the excessive production of NO caused by inflammation, thus inhibiting the increase of iNOS activity in gastric mucosa, while increasing the activity of constitutive NOS (cNOS), inhibiting lymphocyte infiltration in gastric mucosa tissue and playing a protective role in gastric mucosa [39]. The preventive effect of AJWTF on gastric injury may also be due to this mechanism.

## 5. Conclusions

In this study, AJWTFs were used to treat mice with gastric injury, and corresponding indexes of serum and gastric tissues were determined. The results indicated that AJWTFs could (i) significantly repair the abnormal indexes caused by gastric injury, (ii) regulate oxidative stress in serum and gastric tissues to achieve close to normal levels, and (iii) control the expression of related genes in gastric tissues to reach almost normal levels, thereby restoring the effect of gastric injury on gastric tissues. The results showed that AJWTFs exerted a positive effect on the repair and prevention of gastric injury, and its intensity depended on AJWTF concentration. With an increase in AJWTF concentration, the preventive effect of AJWTFs on gastric injury was enhanced. Moreover, 200 mg/kg AJWTF could achieve nearly the same effect as that of ranitidine. Therefore, AJWTFs can be used and developed as active substances to prevent alcohol-induced gastric injury, particularly as functional foods to counter the effect of alcohol or for the repair of chronic alcohol-induced gastric injury.

## Figures and Tables

**Table 1:** Sequences of primers used in this study.

Gene Name	Sequence
nNOS	Forward: 5’-GAATACCAGCCTGATCCATGGAA-3’
	Reverse: 5’-TCCTCCAGGAGGGTGTCCACCGCATG-3’
eNOS	Forward: 5’-TCAGCCATCACAGTGTTCCC-3’
	Reverse: 5’-ATAGCCCGCATAGCGTATCAG-3’
iNOS	Forward: 5’-GTTCTCAGCCCAACAATACAAGA-3’
	Reverse: 5’-GTGGACGGGTCGATGTCAC-3’
Cu/Zn–SOD	Forward: 5′-AACCAGTTGTGTTGTCAGGAC-3′
	Reverse: 5′-CCACCATGTTTCTTAGAGTGAGG-3′
Mn–SOD	Forward: 5’-CAGACCTGCCTTACGACTATGG-3’
	Reverse: 5’-CTCGGTGGCGTTGAGATTGTT-3’
CAT	Forward: 5’-GGAGGCGGGAACCCAATAG-3’
	Reverse: 5’-GTGTGCCATCTCGTCAGTGAA-3’
GAPDH	Forward: 5’-AGGTCGGTGTGAACGGATTTG-3’
	Reverse: 5’-GGGGTCGTTGATGGCAACA-3’

nNOS: Neuronal nitric oxide synthase; eNOS: Endothelial nitric oxide synthase; iNOS: Inducible nitric oxide synthase; Cu/Zn–SOD: Copper/zinc superoxide dismutase; Mn–SOD: Manganese superoxide dismutase; CAT: Catalase; GAPDH: Glyceraldehyde-3-phosphate dehydrogenase.

**Table 2:** Volume and pH of gastric fluid in mice from each group (*n* = 10).

Group	Gastric Juice Volume (mL)	Gastric Juice pH
Normal	0.01 ± 0.01 ^b^	3.90 ± 0.17 ^a^
Model	0.22 ± 0.03 ^a^	1.91 ± 0.11 ^d^
Ranitidine	0.13 ± 0.03 ^ab^	2.52 ± 0.12 ^b^
AJWTF-L	0.19 ± 0.04 ^ab^	2.10 ± 0.15 ^c^
AJWTF-H	0.15 ± 0.02 ^ab^	2.31 ± 0.14 ^bc^

Values presented are the means ± standard deviation. ^a–^^d^ Mean values with different letters in the same column indicate significant difference (*p* < 0.05) according to Duncan’s multiple-range test. Ranitidine: Ranitidine at 50 mg/kg body weight (b.w.) by gavage; AJWTF-L: Anji white tea flavonoids at 100 mg/kg b.w. by gavage; AJWTF-H: Anji white tea flavonoids at 200 mg/kg b.w. by gavage.

**Table 3:** Degrees of gastric injury in mice of each group (*n* = 10).

Group	Area of Gastric Injury (mm^2^)	Inhibitory Rate of Gastric Injury (%)
Normal	0.00 ± 0.00 ^e^	100 ± 0.00 ^a^
Model	20.56 ± 5.29 ^a^	-
Ranitidine	1.98 ± 0.62 ^d^	90.37 ± 5.86 ^b^
AJWTF–L	13.44 ± 1.57 ^b^	34.63 ± 3.48 ^d^
AJWTF–H	3.76 ± 1.65 ^c^	81.71 ± 2.12 ^c^

Values presented are the means ± standard deviation. ^a–^^e^ Mean values with different letters in the same column indicate significant difference (*p* < 0.05) according to Duncan’s multiple-range test. Ranitidine: Ranitidine at 50 mg/kg body weight (b.w.) by gavage; AJWTF–L: Anji white tea flavonoids at 100 mg/kg b.w. by gavage; AJWTF–H: Anji white tea flavonoids at 200 mg/kg b.w. by gavage.

**Table 4:** Serum levels of superoxide dismutase (SOD), glutathione (GSH), and malondialdehyde (MDA) in mice from each group (*n* = 10).

Group	SOD (U/mL)	GSH (mg/L)	MDA (nmol/mL)
Normal	155.595 ± 3.669 ^a^	19.112 ± 0.890 ^a^	6.000 ± 0.003 ^c^
Model	61.732 ± 11.933 ^e^	8.8789 ± 0.261 ^e^	10.667 ± 1.670 ^a^
Ranitidine	137.679 ± 8.663 ^b^	18.209 ± 0.987 ^b^	6.444 ± 1.031 ^ab^
AJWTF–L	95.032 ± 7.944 ^d^	10.233 ± 0.952 ^d^	9.222 ± 0.956 ^a^
AJWTF–H	113.532 ± 18.779 ^ac^	13.243 ± 0.426 ^c^	7.556 ± 0.685 ^ab^

Values presented are the means ± standard deviation. ^a–^^e^ Mean values with different letters in the same column indicate significant difference (*p* < 0.05) according to Duncan’s multiple-range test. Ranitidine: Ranitidine at 50 mg/kg body weight (b.w.) by gavage; AJWTF-L: Anji white tea flavonoids at 100 mg/kg b.w. by gavage; AJWTF-H: Anji white tea flavonoids at 200 mg/kg b.w. by gavage.

**Table 5:** Superoxide dismutase (SOD) and glutathione (GSH) activities and malondialdehyde (MDA) level in stomach tissues of mice from each group (*n* = 10).

Group	SOD (U/mg protein)	GSH (mg/g protein)	MDA (nmol/mg protein)
Normal	62.42 ± 2.880 ^a^	10.106 ± 0.184 ^a^	0.460 ± 0.069 ^d^
Model	30.17 ± 0.820	3.956 ± 0.577 ^d^	0.753 ± 0.059 ^a^
Ranitidine	60.42 ± 2.220 ^a^	9.232 ± 0.913 ^a^	0.486 ± 0.059 ^cd^
AJWTF–L	40.33 ± 2.260 ^c^	6.196 ± 0.752 ^c^	0.691 ± 0.040 ^b^
AJWTF–H	52.11 ± 3.780 ^b^	8.119 ± 0.773 ^b^	0.582 ± 0.065 ^c^

Values presented are the means ± standard deviation. ^a–^^d^ Mean values with different letters in the same column indicate significant difference (*p* < 0.05) according to Duncan’s multiple-range test. Ranitidine: Ranitidine at 50 mg/kg body weight (b.w.) by gavage; AJWTF-L: Anji white tea flavonoids at 100 mg/kg b.w. by gavage; AJWTF-H: Anji white tea flavonoids at 200 mg/kg b.w. by gavage.
